# Efficacy of Ultrasound-guided Radiofrequency Ablation of Parathyroid Hyperplasia: Single Session vs. Two-Session for Effect on Hypocalcemia

**DOI:** 10.1038/s41598-020-63299-8

**Published:** 2020-04-10

**Authors:** Zeng Zeng, Cheng-Zhong Peng, Ji-Bin Liu, Yi-Wen Li, Hong-Feng He, Qiao-Hong Hu, Bo Lin, Xiao-Gang Shen

**Affiliations:** 1 Department of Ultrasound, Zhejiang Provincial People’s Hospital, Hangzhou Medical College, Hangzhou, Zhejiang China; 2 Department of Radiology, Thomas Jefferson University Hospital, Philadelphia, USA; 3 Department of Nephrology, Zhejiang Provincial People’s Hospital, Hangzhou Medical College, Hangzhou, Zhejiang China

**Keywords:** Developmental biology, Diseases

## Abstract

To evaluate safety and efficacy of one- vs. two-session radiofrequency ablation (RFA) of parathyroid hyperplasia for patients with secondary hyperparathyroidism (SHPT) and to compare the outcome of both methods on hypocalcemia. Patients with secondary hyperparathyroidism underwent ultrasound guided RFA of parathyroid hyperplasia. Patients were alternately assigned to either group 1 (n = 28) with RFA of all 4 glands in one session or group 2 (n = 28) with RFA of 2 glands in a first session and other 2 glands in a second session. Serum parathyroid hormone (PTH), calcium, phosphorus and alkaline phosphatase (ALP) values were measured at a series of time points after RFA. RFA parameters, including operation duration and ablation time and hospitalization length and cost, were compared between the two groups. Mean PTH decreased in group 1 from 1865.18 ± 828.93 pg/ml to 145.72 ± 119.27 pg/ml at 1 day after RFA and in group 2 from 2256.64 ± 1021.72 pg/ml to 1388.13 ± 890.15 pg/ml at 1 day after first RFA and to 137.26 ± 107.12 pg/ml at 1 day after second RFA. Group 1’s calcium level decreased to 1.79 ± 0.31 mmol/L at day 1 after RFA and group 2 decreased to 1.89 ± 0.26 mmol/L at day 1 after second session RFA (P < 0.05). Multivariate analysis showed that hypocalcemia was related to serum ALP. Patients with ALP ≥ 566 U/L had lower calcium compared to patients with ALP < 566 U/L up to a month after RFA (P < 0.05). Group 1’s RFA time and hospitalization were shorter and had lower cost compared with Group 2. US-guided RFA of parathyroid hyperplasia is a safe and effective method for treating secondary hyperparathyroidism. Single-session RFA was more cost-effective and resulted in a shorter hospital stay compared to two sessions. However, patients with two-session RFA had less hypocalcemia, especially those with high ALP.

## Introduction

Secondary hyperparathyroidism (SHPT) commonly occurs in patients with end stage renal disease (ESRD) when low calcium levels and high phosphorus levels over time stimulate increased PTH secretion^1–3^. SHPT increases the risk for osteoporosis and kidney stones, as well as for parathyroid hyperplasia, a condition that can cause mental abnormalities, renal osteodystrophy, calcific uremic arteriolopathy, vascular calcification, muscle spasms and even lead to respiratory or cardiac arrest^4^.

Treatment for SHPT includes vitamin D sterols, intravenous vitamin D analogs and cinacalcet^5–10^ to improve biochemical profiles and other surrogate markers^11^. Patients with severe SHPT may be candidates for parathyroidectomy (PTX), which increases long-term survival and reduces the risk of fracture in ESRD patients^12^. However, hyperparathyroidism recurs in up to 30% of patients treated with PTX due to incomplete excision of all hyperplasic parathyroid glands^13,14^. Meanwhile, PTX can potentially result in permanent hypoparathyroidism if the parathyroid glands are over-excised^15^.

Recently, various percutaneous ablation methods have been used in treating patients with hyperparathyroidism, including ethanol injection^16^, acetic acid injection^17^, laser ablation^18^, high-intensity focused ultrasound (HIFU) treatment^19^, microwave ablation^20–22^ and radiofrequency ablation (RFA)^23–25^. Ethanol injection is considered a minimally invasive treatment for SHPT, but the 1-y recurrence rate is 80% due to incomplete ablation^26^. Laser ablation use in parathyroid gland diseases has mainly focused on primary hyperparathyroidism caused by parathyroid gland adenoma^27–29^. Meanwhile HIFU is not yet a clinically viable option because the equipment needed is not widely available. Both microwave ablation and RFA are considered minimally invasive techniques with similar a principle and mechanism for local tissue coagulation. We have used RFA to treat parathyroid hyperplasia in patients with SHPT and to reduce serum PTH, calcium, and phosphorus levels. Complications from RFA, such as nerve injury, hemorrhage, infection, trauma and fever, are fewer and recovery is shorter compared with surgery^30^.

RFA has been shown to be particularly feasible for optimally treating SHPT patients with chronic kidney disease (CKD) whose PTH level is > 800 pg/mL^31^. In these patients, both under- and over-treatment can result in serious complications. In SHPT overtreatment, parathyroid gland inhibition can lead to low PTH levels and cause dynamic bone disease and even increased mortality. However, SHPT undertreatment can allow high PTH levels to induce a series of systemic disorders related to abnormal mineral metabolism of the bone, which seriously affects quality of life and can be life threatening. Thus, serum PTH levels must be maintained at a reasonable level for patients with CKD on dialysis^32–34^. For patients with ESRD, intact PTH levels should be 150–300 pg/mL, according to the National Kidney Foundation Kidney Disease Outcomes Quality Initiative^35^. Because of variability in PTH measurement, the 2009 Kidney Disease Improving Global Outcomes (KDIGO) guidelines suggested that patients with CKD-Stage 5D should control their PTH levels between 2- and 9-fold of the upper limit of normal^2^.

Our previous studies demonstrated that ultrasound (US)-guided RFA of parathyroid hyperplasia is effective for treating SHPT^30^. However, the severe hypocalcemia has been critical issues after thermal ablation, which may be related to the timing and sessions of the treatment. Many complications will occur if in severe hypocalcemia, such as neuromuscular irritability, seizures, myocardial dysfunction and pulmonary abnormalities^36^. Recent research hypotheses that because of trasnsient severe hypocalcemia, the mortality rate would be higher^37^. Thus, maintaining calcium in a normal level becomes very important. Few studies showed that the preoperative alkaline phosphatase (ALP) level and the PTH level could be used to predict the calcium level after treatment as well as the RFA modes^38^. The purpose of current study was to evaluate the safety and efficacy of one-session vs. two-session RFA of parathyroid hyperplasia in patients with SHPT and to compare the effect and outcome of both methods on hypocalcemia.

## Materials and Methods

### Patients

**Figure 1:**
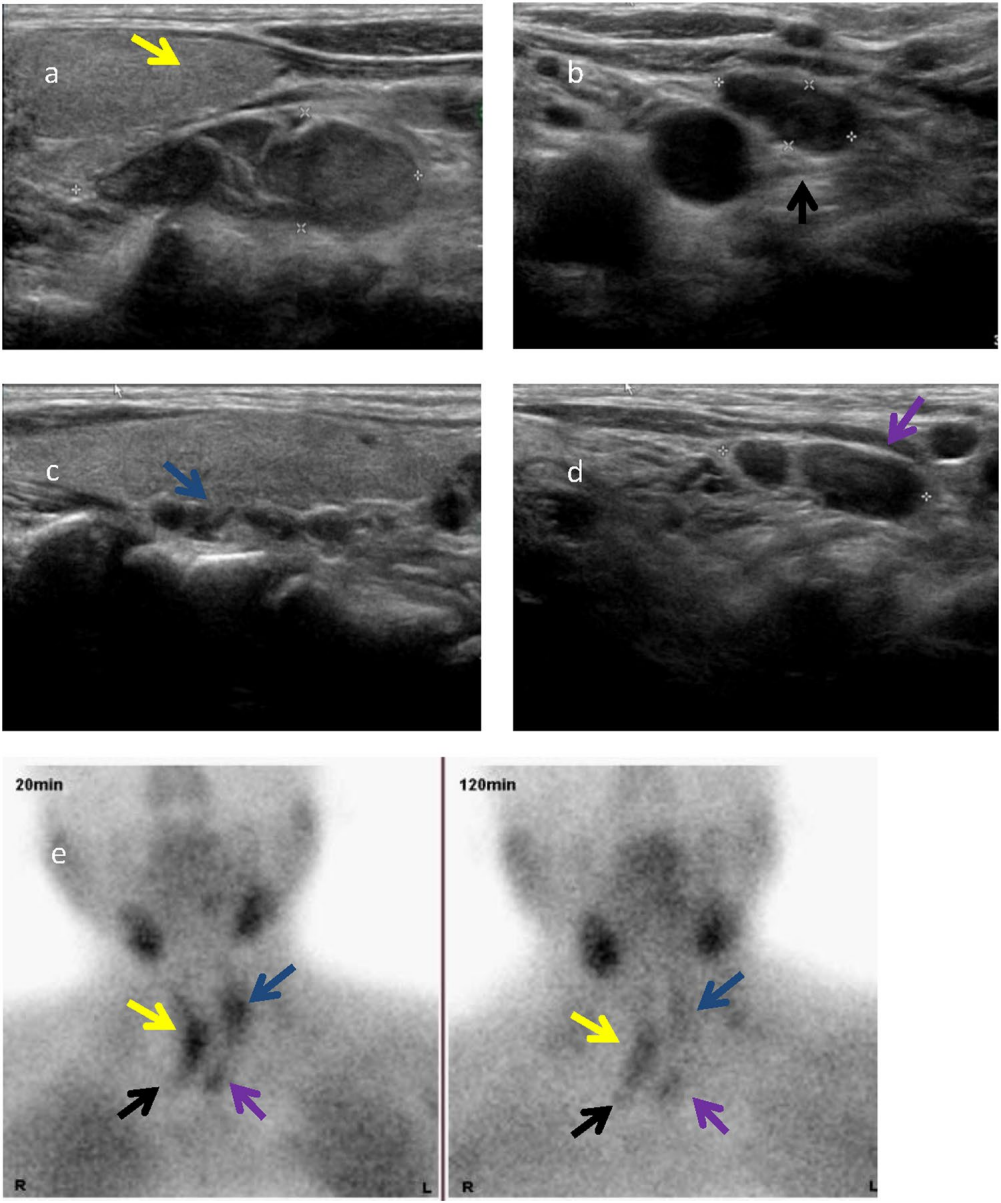
Ultrasound found 4 hyperplastic parathyroid nodules in a patient with secondary hyperparathyroidism (SHPT) and confirmed by ECT. (**a**) The middle and lower right parathyroid gland (yellow arrow). (**b**) The lower right parathyroid gland (black arrow). (**c**) The upper left parathyroid gland (blue arrow). (**d**) The lower left parathyroid gland (purple arrow). (**e**) Emission computed tomography (ECT) coronal image shows that nodule has concentration of radioactivity in the initial phase (20 min) and late phase (120 min) of 99 m Tc-sestamibi sequence.

This study was approved by the ethical and scientific review board of Zhejiang Provincial People’s Hospital. Written informed consent was obtained from all patients enrolled in this study. We confirm that all methods were performed in accordance with the relevant guidelines and regulations. From January 2015 to June 2018, we retrospectively studied 56 patients (22 men, 34 women) who were diagnosed as secondary hyperparathyroidism caused by CKD. All 56 patients had dialysis history, including 21 patients with the peritoneal method and 35 patients with hemodialysis. The inclusion criteria were: (1) PTH ≥ 800 ng/ml; (2) 4 hyperplastic parathyroid glands found by ultrasound (Fig. 1); (3) uncontrollable SHPT using medication treatment; and (4) no severe bleeding disorders, cardiac insufficiency, or uncontrollable hypertension. The exclusion criteria were: (1) abnormal vocal cord movement by laryngoscopy; (2) abnormal coagulation function tests (prothrombin time > 25 seconds, prothrombin activity < 40% and platelet count <100 × 10^9^/L); (3) refusing RFA by patient; (4) severe cardiopulmonary dysfunction; and (5) When hoarseness occur after the first session ablation.

Patients were alternately assigned into 2 groups: group 1 (n = 28) with RFA of all 4 glands in one session and group 2 (n = 28) with RFA of 2 glands in a first session and 2 glands in a second session 1 week later.

### Laboratory tests and follow-up

All patients’ demography, primary disease, dialysis history, and clinical symptoms were recorded. Blood tests of PTH, calcium, and phosphorus were conducted 24 hours before RFA, and on day 1, day 3, 1 week, and 1 month after RFA. Normal ranges were defined as: PTH 11–62 pg/mL, serum calcium 2.11–2.52 mmol/L and serum phosphorous 0.85–1.51 mmol/L. Clinically, serum calcium <1.8 mmol/L is considered as obviously low calcium, warranting treatment with intravenous calcium supplementation. Thus, blood calcium <1.8 mmol/L was used as the standard for remarkable low calcium limitation. When blood calcium is lower than 2 mmol/L but higher than 1.8 mmol/L, oral calcium supplementation is given and otherwise, intravenous calcium will be administered. Previous studies showed that after parathyroidectomy bone resorption markers decreased significantly after 24 hours while bone formation markers changed minimally^39,40^. In this study, the lowest calcium level on the first day after RFA was used to decide whether hypocalcemia or not in order to avoid the effect of active calcium supplementation given later. Because alkaline phosphatase (ALP) is a marker for bone formation and could not change noticeably within 1 to 3 days after RFA, measurement of bone ALP was performed before RFA and at 1 week and 1 month after RFA.

### US-Guided RFA

**Figure 2:**
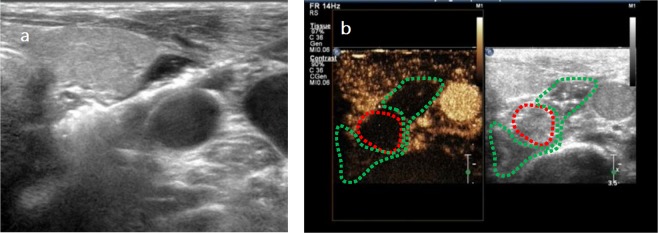
(**a**) The radiofrequency ablation (RFA) electrode reached the location on the back of the parathyroid gland. (**b**) Contrast enhanced ultrasound (CEUS) was used to monitor the radiofrequency ablation (RFA) procedures, a buffering zone (green dotted line) around the hyperplastic parathyroid (red dotted line).

RFA procedure was performed by single doctor (CZP) with 22 years of experience in interventional ultrasound and 10 years of experience in RFA treatment of thyroid and parathyroid nodules. Patients were placed in a supine position with their necks elevated using a pillow under their shoulders. With routine disinfection and draping as well as 2% lidocaine local anesthesia, US-guided RFA was performed using grey-scale imaging with a iU22 US scanner and a high-frequency linear probe (L12-5) (Philips, The Netherlands) while contrast-enhanced ultrasound (CEUS) with a high-frequency linear probe (L9-3) was used to monitor the RAF procedures (Fig. 2). Thermal ablation of hyperplastic parathyroid glands was conducted using a radiofrequency generator with an 18-gauge monopolar internally cooled electrode (VIVA; STARmed, Goyang, Korea). The radiofrequency electrode had a 7-cm length shaft with a 0.7-cm active tip and was cooled by a water circulation pump (VIVA pump; STARmed).

**Figure 3:**
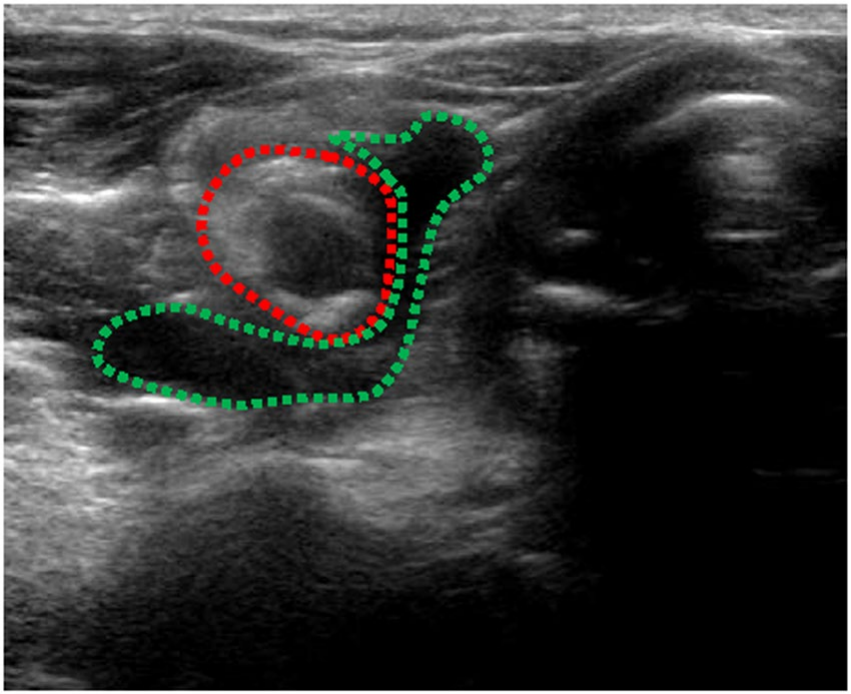
We injected 15 ml 0.9% sterile saline solution to create a buffering zone (green dotted line) around the hyperplastic parathyroid (red dotted line) in order to establish a liquid isolation zone >1 cm in depth between the parathyroid and adjacent structures.

In order to avoid thermal injury of adjacent structures, a buffering zone was created by injection of 10–30 mL of 0.9% sterile saline solution around the hyperplastic parathyroid to establish a liquid isolation zone>1 cm in depth between the parathyroid and adjacent structures (Fig. 3). Then, the RFA electrode was inserted and deployed 1 mm away from the back of the parathyroid gland under real-time US guidance. Thermal ablation effect showed a hyperechoic area within the coagulated tissue. The entire targeted glands were ablated by moving the electrode step-by-step until coagulation was complete. During the procedure and after ablation of each gland, the operator attempted to ask the patients to evaluate their voice. If voice hoarseness happened, the procedure was stopped. If not, the procedure continued.

### Statistical analysis

Data analysis was performed using the SPSS software (SPSS for Windows 22.0, SPSS, Chicago, IL) and Graphpad Prism version 4.03 (California, USA). Continuous variables were expressed as the mean ± standard deviation. Multivariate logistical regression analysis was used to screen independent features for the occurrence of low calcium. Continuous variables were analyzed by using the Student *t* test. P < 0.05 was used to define significance.

## Results

Patient demographics and clinical features of appear in Table 1. For group 1 patients, the maximum diameter of the glands ranged from 0.7 cm to 2.9 cm (mean, 1.4 cm ± 0.5). For group 2 patients, the maximum diameter of the glands ranged from 0.8 cm to 2.5 cm (mean, 1.6 cm ±0.4). Table 2 shows the results of the laboratory test analysis. In group 1, mean PTH level decreased from 1865.18 ± 828.93 pg/ml to 145.72 ± 119.27 pg/ml at 1 day after RFA and to 289.04 ± 197.99 pg/ml at 1 month after RFA. In group 2, mean PTH level decreased from 2256.64 ± 1021.72 pg/ml to 1388.13 ± 890.15 pg/ml at 1 day after first-session RFA and then rebounded to 1625.92 ± 1129.43 pg/ml at 7 days later. Group 2 mean PTH decreased sharply and rapidly from 1677.66 ± 1012.36 pg/ml to 137.26 ± 107.12 pg/ml at 1 day after second-session and to 230.92 ± 190.23 pg/ml at 1 month after second-session RFA. The mean PTH level in group 1 and group 2 after complete RFA was not statistically significant (P > 0.05).

**Table 1:** Clinical features of group 1 and group 2 patients with SHPT.

Features	Group 1 (N = 28)	Group 2 (N = 28)
**Sex**		
Male	7	15
Female	21	13
Age	53.50 ± 10.06	45.53 ± 12.93
**Dialysis Method**		
Peritoneal	11	10
Hemodialysis	17	18
Dialysis Duration(months)	93.00 ± 28.67	90.66 ± 26.98

**Table 2:** Laboratory tests of group 1 and group 2 patients before and after RFA.

Laboratory tests	Group 1		Group 2(1^st^ RFA)		Group 2(2^nd^ RFA)	
	Before RFA	After RFA(day 1)	Before RFA	After RFA(day 1)	Before RFA	After RFA(day 1)
PTH, pg/ml	1865.18 ± 828.93	145.72 ± 119.27	2256.64 ± 1021.72	1388.13 ± 890.15	1677.66 ± 1012.36	137.26 ± 107.12
Calcium, mmol/L	2.59 ± 0.17	1.79 ± 0.31	2.53 ± 0.19	2.22 ± 0.23*	2.31 ± 0.19	1.89 ± 0.26*
Phosphorus, mmol/L	2.08 ± 0.32	1.57 ± 0.44	2.10 ± 0.58	1.89 ± 0.57	1.70 ± 0.32	1.28 ± 0.56
ALP, U/L	488.03 ± 439.80	488.04 ± 447.87	612.89 ± 550.13	590.46 ± 556.55	662.39 ± 527.13	690.47 ± 528.03

P^*^<0.05 for group 2 compared with group 1 after first RFA on day 1.

Normal ranges: PTH 11–62 pg/mL, Calcium 2.11–2.52 mmol/L and Phosphorous 0.85–1.51 mmol/L.

**Figure 4:**
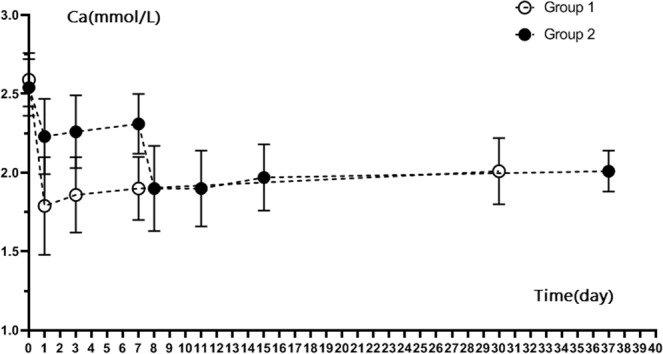
Calcium level of Group 1 and Group 2 patients after RFA.

Calcium decreased quickly after RFA both in group 1 and group 2 patients (Fig. 4). 27 patients (27/28, 96.4%) in Group 1 had hypocalcemia on the 1st day after RFA. In Group 2, 24 patients (24/28, 85.7%) had hypocalcemia on the 1st day after second session of RFA. The calcium line of group 1 was below group 2. The mean calcium level in group 1 decreased quickly from 2.59 ± 0.17 mmol/L before RFA to 1.79 ± 0.31 mmol/L at day 1 after RFA. The mean calcium level in group 2 also decreased from 2.53 ± 0.19 mmol/L before RFA to 2.22 ± 0.23 mmol/L at day 1 after first-session RFA and to 1.89 ± 0.26 mmol/L at day 1 after second-session RFA. Calcium level between group 2 after second RFA and group 1 at day 1 had significance (P <0.05). In group 1, 17 patients had oral calcium supplementation, 10 patients had intravenous calcium administration and 1 patients had no need to have calcium supplement. In group 2 after 2^nd^ RFA, 14 patients had oral calcium supplementation, 10 patients had intravenous calcium administration and 4 patients had no need to have calcium supplement. After active calcium supplementation therapy, calcium levels gradually increased.

**Figure 5:**
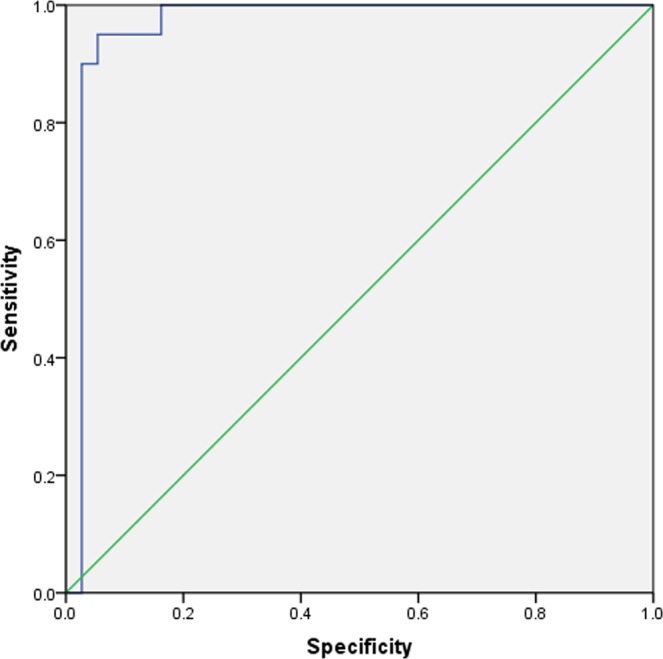
ROC curve of alkaline phosphatase (ALP) shows area under the curve of 0.965, sensitivity of 95.0% and specificity of 94.6% using alkaline phosphatase (ALP) cutoff point as 566 mmol/L.

Table 3 shows the binary logistic regression analysis of PTH and ALP values (before RFA) for occurrence of hypocalcemia. Although both serum PTH and ALP presented as significant factors for the occurrence of hypocalcemia, ALP’s effects on hypocalcemia occurrence was significant (P < 0.05) compared to PTH’s effects (P > 0.05). When the cutoff point of ALP level was set as 566 U/L, an area under the ROC curve was 0.965, Youden index of 0.896, sensitivity of 95.0% and specificity of 94.6% (Fig. 5).

**Table 3:** Binary logistic regression analysis of PTH and ALP values (before RFA) for occurrence of hypocalcemia.

	β	SE	Wald	P	OR (95%CI)
Constant	−2.728	2.303	9.351	0.002	
PTH	0.000	0.001	0.823	0.364	1.000(0.999–1.001)
ALP	0.003	0.001	5.316	0.021^*^	1.003(1.001–1.006)

**Figure 6:**
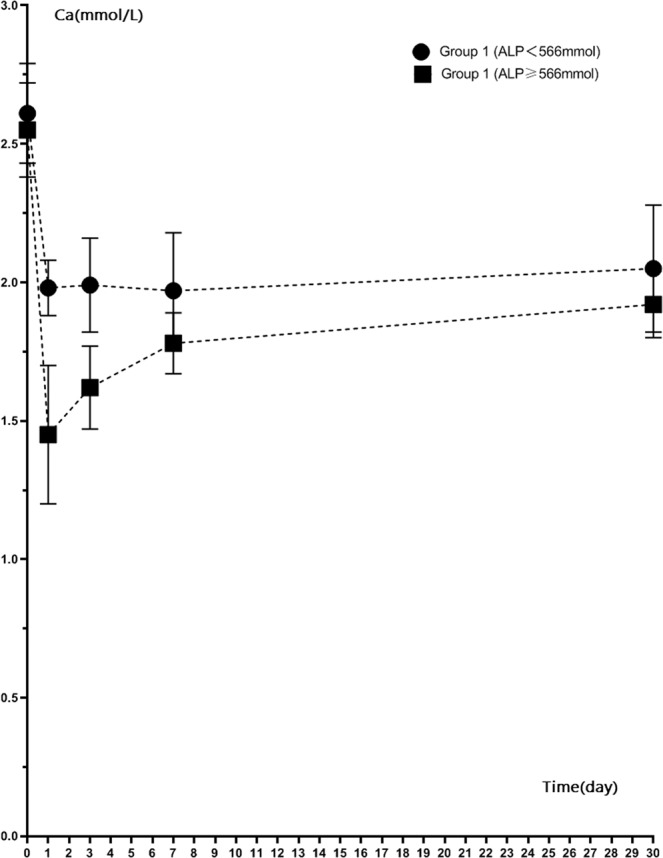
Calcium level of ALP ≥ 566 mmol/L patients and ALP < 566 mmol/L patients in Group 1 after RFA.

**Figure 7:**
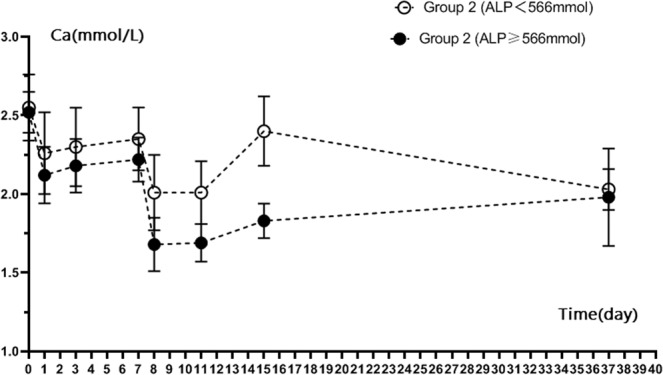
Calcium level of ALP ≥ 566 mmol/L patients and ALP <566 mmol/L patients in Group 2 after RFA.

When we separated patients into a high ALP group (≥566 U/L; 20 patients) and low ALP group (<566 U/L; 36 patients) and measured calcium levels, we found that mean calcium levels at day 1, day 3, 1 week and 1 month after RFA were lower in the high ALP group than in the low ALP group (P < 0.05) (Table 4). Figures 6 and 7 showed the calcium level of group 1 and group 2 patients with different ALP levels. For both two groups, ALP ≥ 566 U/L patients’ calcium level were lower than patients whose ALP < 566 U/L. For the high ALP group, patients with single-session RFA had lower calcium (1.45 ± 0.25 mmol/L) compared to patients with two session RFA 1 day after RFA (1.68 ± 0.17 mmol/L) (P < 0.05). For the low ALP group, patients with single- or two-session RFA had similar low calcium levels (1.98 ± 0.10 mmol/L vs. 2.00 ± 0.27 mmol/L respectively, P > 0.05).

**Table 4:** Mean calcium values before and after RFA in high ALP (>566 U/L) and low ALP (<566 U/L) patients.

Calcium, mmol/L	Low ALP group (N = 36)	High ALP group (N = 20)	P Value
Before RFA	2.40 ± 0.20	2.38 ± 0.22	0.120
Day 1 after RFA	2.03 ± 0.36	1.56 ± 0.24	0.000
Day 3 after RFA	2.12 ± 0.40	1.66 ± 0.15	0.000
1 week after RFA	1.98 ± 0.17	1.80 ± 0.11	0.000
1 month after RFA	2.16 ± 0.19	1.94 ± 0.13	0.040

We also found differences in procedure cost, RFA time, total operation time, and hospitalization time for group 1 and group 2 patients (Table 5). Compared to group 1, costs for the patients in group 2 were higher ($2484.39 ± 199.68 vs. $4658.16 ± 349.37 respectively, P < 0.05) and hospitalization was longer (8.32d ± 1.61 and 15.93d ± 1.60, respectively, P < 0.05). Mean operation duration was 90.53 min±10.31 for group 1 and 100.74 min±8.9 for group 2 of two sessions. The mean RFA time was 225.85 sec±41.85 for group 1 and 214.52 sec±20.47 group 2 for two sessions.

**Table 5:** Hospital stay, cost of RFA, ablation time, and operation duration in group 1 and 2 patients.

	Group 1	Group 2	P Value
Hospital stay	8.32d ± 1.61	15.93d ± 1.60	0.000
Cost of RFA	2484.39$ ± 199.68	4658.16$ ± 349.37	0.000
Ablation time	225.85 sec ± 41.85	214.52 sec ± 20.47 (two sessions)	
Operation duration	90.53 min ± 10.31	100.74 min ± 8.9 (two sessions)	

All patients tolerated the procedures with no major complications. All patients had mild swallowing and variable pain after RFA that disappeared gradually in a few days. One patient in the two-session ablation group developed vocal hoarseness after second ablation and recovered within 70 days.

## Discussion

In previous study, Zhou *et al*. demonstrated the feasibility and efficacy of microwave ablation of parathyroid glands and showed that serum PTH and calcium decreased significantly after MWA in patients with SHPT^41^. In current study, we used US-guided RFA to treat parathyroid hyperplasia in 56 patients with SHPT caused by CKD. Our study showed PTH decreased significantly after RFA for patients in both the one-session and two-session groups. These decreased PTH levels remained decreased and within accepted Kidney Disease Improving Global Outcomes limits^2^ in most of the patients (52/56) at 1 month after RFA, which indicated that RFA can inactivate hyperplastic parathyroid glands and achieved the desired therapeutic effect.

Hypocalcemia is the most common complication after parathyroid resection surgery. In addition to symptoms such as limb numbness and cramps, hypocalcemia can also cause severe complications such as bronchospasm, gastrointestinal spasm, and arrhythmia. Therefore, close postoperative monitoring of blood calcium and timely treatment are critical and necessary. In general, blood calcium should be monitored 2–4 times a day after surgery. When blood calcium is lower than 2.11 mmol/L but higher than 1.8 mmol/L, oral calcium supplementation is given; when blood calcium is lower than 1.8 mmol/L, intravenous calcium supplementation is given. Hypocalcemia is also a common complication of thermal ablation. Our study shows hypocalcemia occurred on the first day after RFA. When hypocalcemia occurred after RFA, active calcium supplementation therapy was effective to gradually correct the calcium level.

PTH has many target organs such as bones, kidneys and the intestinal tract. Because PTH can bind to its receptors on osteoclasts and stimulates bone resorption, it leads to the release of calcium into the blood^42^. In fact, even small changes of serum ionized calcium (lower than 0.1 mmol/L) can cause obvious changes in PTH^43,44^. The effect of low level PTH on bone is mainly osteogenesis and the effect of high level PTH on bone is both osteogenesis and osteoclast. High PTH has greater effects on osteoclast than osteogenesis^20,45^. Thus, patients with SHPT have high serum PTH and high metabolism of bone transport^30^. With complete ablation of hyperplastic parathyroids, serum PTH can decrease rapidly alone with osteoclast activity decreased or disappeared in a short time, and then osteoblastic activity continues. Therefore, in order to keep normal bone metabolism, the components of calcium and phosphorus in the blood must reach the level that the process of bone formation can lead to a rapid decrease of serum calcium and phosphorus levels in a short period of time, a process known as bone starvation^46^. Hypocalcemia after parathyroid RFA or surgery is related to bone starvation.

ALP is an indicator of osteogenesis to some extent, it reflects the high transport state of bone metabolism. Therefore, the higher the ALP, the higher bone transport state, and thus the higher the incidence of hypocalcemia after RFA. Our study found that the low calcium level of patients after ablation was closely and positively correlated with the preoperative serum ALP level. Higher ALP levels before ablation will cause more obvious postoperative hypocalcemia. It was similar with observations in recent decades, owing to the high values of ALP, patients may had higher risk for bone fractures and mortality due to hypocalcemia^47,48^. When the calcium level <1.8 mmol/L was defined as a standard of obvious low calcium, patients with ALP cutoff point at 566 mmol/L presented marked hypocalcemia with sensitivity of 95.0% and specificity of 94.6%. During follow-up 1 month after RFA, calcium levels before ablation were significantly lower in patients with ALP > 566 U/L than in patients with ALP < 566 U/L (1.94 ± 0.13 mmol/L vs. 2.16 ± 0.19 mmol/L) (P = 0.40). Our current study also found that the effects of single-session RFA and two-session RFA on hypocalcemia were different in patients depending on their ALP levels. For those patients with ALP of 566 mmol/L or higher, hypocalcemia was more pronounced in patients with single-session RFA compared with patients with two-sessions at day 1 after RFA (1.45 ± 0.25 mmol/L vs. 1.65 ± 0.20 mmol/L, P = 0.020). For those patients with ALP lower than 566 mmol/L, single-session group and two-session group calcium levels were similar (1.98 ± 0.10 mmol/L vs. 2.04 ± 0.30 mmol/L, P = 0.580). This finding implied that a single session of RFA in patients with high ALP was more likely to cause severe hypocalcemia, while two sessions of RFA was more likely to cause mild hypocalcemia. The reason may be related to the step-by-step descent of PTH, with fractional ablation of the parathyroids in two sessions alleviating bone starvation.

Parathyroid glands are located in the area of the so-called cervical dangerous triangle. The local anatomy of this region has complex structures and the glands are closely adjacent to the recurrent laryngeal nerve. Thermal injury of the recurrent laryngeal nerve can cause voice change during the local coagulation of parathyroid glands. It is one of the most serious complications reported after RFA of both benign thyroid nodule and recurrent thyroid cancer, according to a study by Baek *et al*.^49^. Most patients with thermal damage of the nerve recover within 2 to 3 months after RFA. In the current study, two patients (one in single-session RFA and another in two-session RFA) developed hoarseness after ablation, and they both recovered within 70 days. As described in our previous study, a fluid spacing ablation method and moving-shot technique with 1-mm spare margin of the glands can avoid thermal injury of carotid artery, esophagus, trachea and other adjacent structures, especially for recurrent laryngeal nerves^30^. There were no other severe complications during RFA or within the follow-up period in this study. Both our current and previous studies demonstrated that high resolution US-guided RFA of hyperplastic parathyroid for treatment of patients with SHPT is safe and feasible by using either single- or two-session methods.

There were limitations of this study. First, the number of patients enrolled in our study was relatively small and more patients and long-term follow-up are needed to further study the efficacy and outcome. Second, CEUS was the only method used for monitoring the completion of the ablation. However, a 99mTc-sestamibi sequence may be needed to assess the result of ablation and efficacy of the treatment. Third, this study design cannot conclude how many parathyroid glands should be ablated at each session for optimal balance of ALP and hypocalcemia. At last, this study only followed up for 1 month period and long term follow-up study will conduct in the future.

In conclusion, this study showed that US-guided RFA of parathyroid hyperplasia is a safe and effective method for the treatment of secondary hyperparathyroidism. Hypocalcemia was likely to occur after RFA, with the degree of hypocalcemia related to pre-ablation serum ALP level. The preliminary results demonstrated hypocalcemia was relatively mild in patients with two-session of RFA, especially for patients with baseline ALP > 566 mmol/L. Although single-session RFA of parathyroid hyperplasia was more cost-effective and required a shorter hospital stay, patients with ALP ≥ 566 mmol/L should have two-session RFA in order to avoid severe hypocalcemia after ablation. Patients with ALP < 566 mmol/L could choose either single- or two-session RFA, depending on their condition and on affordability.
